# Education Satisfaction among Pharmacy Graduates in Sweden

**DOI:** 10.3390/pharmacy9010044

**Published:** 2021-02-18

**Authors:** Maria Gustafsson, Andy Wallman, Sofia Mattsson

**Affiliations:** Department of Integrative Medical Biology, Umeå University, SE-901 87 Umeå, Sweden; maria.gustafsson@umu.se (M.G.); andy.wallman@umu.se (A.W.)

**Keywords:** pharmacy education, professional identity, education satisfaction, pharmacists

## Abstract

Education satisfaction is considered important for development of a professional identity and to increase learning. The aim was to investigate and compare education satisfaction over time among pharmacists who have graduated from the pharmacy programs at Umeå University, Sweden. Data concerning education satisfaction were collected using an alumni survey of pharmacists who graduated between 2015 and 2018. This was compared with pharmacists graduating between 2006 and 2014. The majority of the pharmacy graduates were very satisfied with their education (96%) and thought that the programs gave them a clear professional identity (92%). No differences in education satisfaction between graduation years 2015 and 2018 and 2006 and 2014 were found. A majority of the graduates considered that the knowledge and skills acquired during their education were useful in their present job (83%). Of the graduates who thought that the studies gave them a clear professional identity, a higher proportion were satisfied with their job (*p* < 0.001) and thought that their work duties reflected their studies (*p* = 0.005). Exploring education satisfaction may help educators to further develop the education and to better prepare the students for their professional working life.

## 1. Introduction

Many factors have been identified as impacting on students’ education satisfaction such as degree outcome, quality of teaching, and course organization [1,2,3]. Another important factor for education satisfaction among students is the usefulness or relevance of their studies to their job [2]. Furthermore, education satisfaction is important for the ability to adopt a professional identity [4]. Building a common professional identity is complicated by the uncertainty and transformation of the professional role of pharmacists and the role of pharmacies in health care, resulting in an undefined professional identity both in Sweden and internationally [5,6,7]. Students’ wellbeing may also influence academic satisfaction [8] and educational factors such as workload, learning environment, and meaningfulness of educational activities may in turn affect wellbeing [9]. Students’ satisfaction with their education appears to be important in regard to success and failure to learn [10]. One study, using data from the UK National Student Survey (NSS) that represented data from 2.3 million full-time students, found that there has been an increase in overall education satisfaction over the decade 2007 to 2016 [11]. Measuring the students’ level of satisfaction post-graduation is considered to be an advantage because the students may have a greater appreciation of the value of their education after they have started their professional career [11].

In Sweden, not only has community pharmacy undergone changes because of the re-regulation of the pharmacy market, which has consequently become more commercialized, but primary care is also undergoing changes with employment of more pharmacists, for example [5,12,13]. Pharmacists working in primary care conduct for example medication reviews at nursing homes. A recent study described the community pharmacies in Sweden as being at a crossroad; either continuing to focus on retail business or becoming more integrated in the health care chain [12]. The changes within the pharmacy marketplace demands on pharmacy education in order to prepare students for their professional role and job assignments. In total, four universities are offering pharmacy education in Sweden. The Swedish Higher Education Authority is the centralized accrediting body and is responsible for evaluating the quality of higher education and research. As part of the quality assurance of higher education, students have several opportunities throughout their studies to provide feedback on their academic studies and educational activities using course and program evaluations, for example. The pharmacy programs at Umeå University include courses in chemistry, biomedical sciences, pharmacology, pharmacokinetics, pharmacotherapy, pharmaceutics, social pharmacy, and pharmacy practice. The curricula have been similar over the years, but with regular revision to update the course material and implement new educational activities and assessment methods.

In 2015, a questionnaire was sent to all students who had graduated between 2006 and 2014 from the pharmacy programs at Umeå University, Sweden [14]. This study found that most graduates were satisfied with their education. Furthermore, a majority of the graduates agreed or strongly agreed that the knowledge and skills acquired during the education were useful in their current job, and a majority thought that their duties at work reflected their education. To our knowledge, few studies have investigated pharmacy students’ education satisfaction over time. The objective of the present study was to investigate education satisfaction among pharmacists graduating between 2015 and 2018 from the pharmacy programs at Umeå University, Sweden. A second objective was to compare education satisfaction among these graduates with those graduating between 2006 and 2014.

## 2. Materials and Methods

### 2.1. Setting

Umeå University offers three pharmacy programs; a Bachelor of Science in Pharmacy (three years), a Master of Science in Pharmacy (five years), and a Master of Science in Pharmaceutical Science (two years). Graduates who already hold a bachelor’s degree in pharmacy are eligible to apply to the Master of Science in Pharmaceutical Science program in order to pursue a master’s degree. In Sweden, there are two professional degrees within pharmacy; prescriptionists with a bachelor’s degree and pharmacists with a master’s degree. Both prescriptionists and pharmacists work at community pharmacy with dispensing and counselling of medicines. Community pharmacy is the major workplace for prescriptionists, but they may for example also work in hospitals with drug supply and in hospital pharmacies with extemporaneous compounding of medicines. Pharmacists can also work as clinical pharmacists with for example medication reviews. To be able to work in healthcare, both degrees need a registration issued by the National Board of Health and Welfare. Besides working in the healthcare section, pharmacists are also, to a greater extent than prescriptionists, employed in pharmaceutical industry and governments such as the Swedish Medical Products Agency and the Dental and Pharmaceutical Benefits Agency. The term ‘pharmacists’ will be used throughout the paper to describe both degrees. All three programs are web-based and meetings between students and teachers occur primarily online. Online meetings occur 1–2 times each week where the teacher and students discuss different topics depending on the course. Besides this, students also conduct group work where the students meet online to prepare assignments. In addition, the students have access to online material through a virtual learning platform, for example. recorded lectures, animations, and assignments. Some educational activities, for example laboratory work, oral presentations, and role play, take place on campus, approximately 2–4 times each semester. Pharmacy practice at community pharmacy is included in all three programs. Students admitted to the five-year master’s program study together with students admitted to the three-year bachelor program, and students admitted to the five-year master program study together with the students admitted to the two-year program in their two last years. Considering this set up, the programs are closely interconnected and may therefore be combined to examine education satisfaction. As mentioned above, there are four universities in Sweden offering pharmacy education. Umeå University is the only university that offers pharmacy education on both bachelor and master level online with no campus-based alternatives. One other university has an online bachelor program in pharmacy, but this university also has a campus-based program. The two other universities offer campus-based pharmacy programs on both bachelor and master level.

### 2.2. Survey

To study how satisfied the graduates are with their education, a survey was conducted among graduates from the Bachelor of Science in Pharmacy, Master of Science in Pharmacy and Master of Science in Pharmaceutical Science programs at Umeå University. The students graduated between 2015 and 2018, and the survey was sent out in March 2019 to 222 graduates. Of these graduates, 16 had obtained both a bachelor’s and a master´s degree. The survey, together with a postage-paid envelope, was sent by post to the graduates’ address in the university´s administrative register. This register is continuously updated against the Swedish Tax Agency which manages the civil registration of private individuals in Sweden. For graduates without a Swedish address in the register (16 graduates), an e-mail was sent with an invitation to participate in the survey. Of these 16 graduates, two accepted the invitation to participate and the survey was sent to them by post. In total, 94 graduates participated in the survey (response rate 42%). No reminders were sent out. A similar survey investigating education satisfaction had been conducted in 2015 and the present survey was developed based on this previous survey [14]. The students were asked questions about why they chose to study pharmacy at Umeå University, their education, and their attitude towards various educational activities. In addition to questions regarding their education, the graduates were also asked questions about their job and job satisfaction [15].

### 2.3. Data Analysis

Descriptive statistics were used to summarize the data. Table 1 presents the questions used in the present study and how the answers were dichotomized. To compare data regarding questions 1–4 between master’s and bachelor’s graduates, the chi-squared test was used. Furthermore, the groups investigated in 2015 and 2019 were compared regarding questions 5–8. A logistic regression model was constructed for each question to control for age between the two samples. The answers to question 9 are reported as the percentage of graduates choosing each answer. To compare data between 2015 and 2019, the chi-squared test was used. Finally, graduates who were satisfied and not satisfied with their job were compared regarding question 3. Graduates who agreed and disagreed to question 3 were also compared regarding question 7. Data were compared using the chi-squared test.

Results are presented as odds ratios (ORs) with 95% confidence intervals (CIs)**.** A p-value of <0.05 was considered statistically significant. All analyses were conducted using Statistical Package for the Social Sciences (SPSS) for Windows version 26.

### 2.4. Ethics

This type of study does not require ethical committee approval in Sweden. All respondents were given information about the aim of the study and that the data would be treated as strictly confidential and that all answers would be anonymous. By completing and returning the survey, the respondents agreed to that the results could be used for research purposes.

## 3. Results

Table 2 presents the characteristics of the respondents. Overall, one-third of the respondents had graduated from the Master in Pharmacy program and two-thirds from the Bachelor in Pharmacy program. A majority of the graduates were female, and the average age was 35.0 years. Furthermore, a majority of the respondents were born in Sweden and worked in community pharmacy. Among the graduates with a master’s degree, greater diversity was seen regarding their workplace which also included pharmaceutical companies and county councils.

Table 3 presents the respondents’ answers to questions related to education satisfaction. A majority (81.6%) agreed or strongly agreed that they were well prepared to work as a pharmacist after completing the studies and that the education gave them a clear professional identity (92.2%). Furthermore, a majority of the respondents (72.6%) agreed or strongly agreed that their opinions during their education were considered important by the teachers and that they were very satisfied with their education (95.7%). In Table 2, comparison of education satisfaction for master’s and bachelor’s graduates revealed only one difference which related to the question ‘I felt well prepared to work as a pharmacist after completing the program’. Here, a significantly higher percentage of the master’s graduates (93.3%) agreed or strongly agreed with the statement that they felt well prepared to work as a pharmacist compared with the bachelor’s graduates (75.8%) (*p* = 0.042). The other questions did not show any difference between the two groups (master’s degree vs bachelor’s degree) (*p* > 0.05).

The answers for education satisfaction were compared with answers from students graduating between 2006 and 2014 (data collected in a survey in 2015) [14]. A majority of the respondents in the survey in 2019 (graduation years 2015 to 2018) considered that the knowledge and skills acquired during the education were useful in their present job and would recommend the program to a prospective student (Table 4). Furthermore, a majority thought that their duties at work reflected their education and that, if they were to choose again, they would choose pharmacy as a career. There were no differences between the groups (graduation years 2006 to 2014 vs 2015 to 2018) (*p* > 0.05).

There was a significantly higher percentage of graduates who were satisfied with their job (*p* < 0.001) and who thought that their duties at work reflected the program (*p* = 0.005) among those who thought that the education gave them a clear professional identity. This contrasts with those who did not think that the education gave them a clear professional identity.

**Figure 1:**
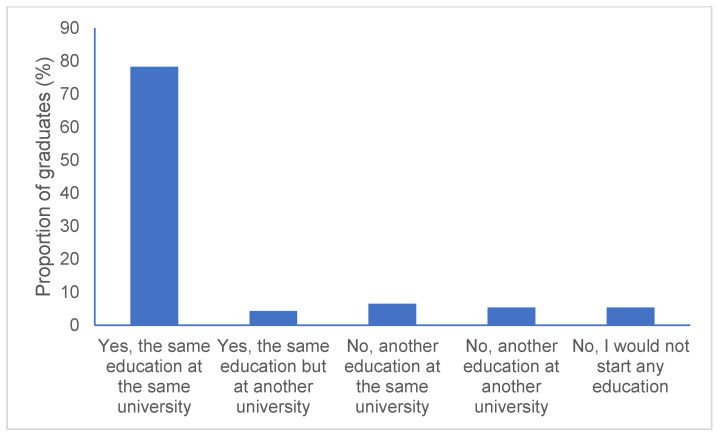
Percentage of graduates answering the different options to the question ‘If I were to choose today, I would start the same education’. Graduation years 2015 to 2018.

Figure 1 shows the results for the question ‘If I were to choose today, I would start the same education’. A majority of the students (78.3%) stated that they would choose the same education at the same university. In the study from 2015 (graduation years 2006 to 2014), 81.8% answered that they would choose the same education at the same university [14]. The difference was not statistically significant (*p* = 0.475).

The survey also asked whether the students, a year or a couple of years after graduation had changed their perception of the value of their education compared to immediately after graduation. Overall, 48% of the graduates answered that they had not changed their perception. Moreover, 32% stated that they were very much more or slightly more positive, whereas 14% stated that they were very much more or slightly more negative. In total, 6% answered that they did not know. The graduates were also able to provide comments to this question.

The graduates were also asked questions regarding various educational activities and whether they felt that these activities were useful after graduation (Table 5). The graduates believed that, in particular, pharmacy practice, lectures, seminars, and problem-based learning were of great or very great usefulness after graduation. However, activities such as laboratory experiments and role play were considered less useful.

The graduates were also asked why they chose to study pharmacy at Umeå University. This was an open-ended question and the results showed that, for most students, the main reason was that the education was offered online. Other reasons included: it was an interesting education; the education had a good reputation; it gave them the possibility to study from a bachelor’s to master’s degree through the Master of Pharmaceutical Sciences program; and it gave them the possibility to work in healthcare.

## 4. Discussion

This study explored education satisfaction among pharmacy graduates who had completed web-based studies. The overall education satisfaction among the graduates participating in the survey was very high; 95.7% agreed or strongly agreed that they were very satisfied with their education. Compared to a study among pharmacy students in Australia, education satisfaction appeared to be higher in the present study [16]. The study was conducted among final year pharmacy students at three universities in Australia and in a survey, the students were asked to rank their satisfaction with their decision to study pharmacy. A majority of the students were “somewhat satisfied” with their choice and a higher proportion of the students stated that they were “not at all satisfied” compared with those who were “extremely satisfied”. A survey from another university in Sweden showed that, three years after graduating from a campus-based Master of Science in Pharmacy program, 46% of the graduates were not satisfied with their choice of studies and would not choose the same studies again [17]. However, direct comparisons may be difficult due to different settings and survey designs.

A majority of the respondents answered that they would choose the same education at the same university if they were to choose again and would recommend the program to a prospective student. These results agree with those from the previous investigation in 2015 [14] and analysis showed no differences between the two groups. Overall, the results of both surveys showed that education satisfaction was high, suggesting that education satisfaction is maintained over a relatively long period of time, i.e., 2006–2018 [14]. Furthermore, students’ satisfaction with their studies has previously been shown to influence their ability to learn [10] indicating that it is important for educators to explore education satisfaction among students and graduates.

A majority of the graduates participating in the survey in 2019 considered that the knowledge and skills acquired during their education were useful in their present job. Moreover, a majority thought that their duties at work reflected their education and that they would, if they were to choose again, choose pharmacy as a career. These results were in agreement with the previous investigation in 2015 [14], and analysis showed no significant differences between the two groups. The results indicate that the programs are able to prepare the students for their professional working life. A majority of the respondents felt that the program gave them a clear professional identity, and that they felt prepared to start work as pharmacists after completing their education. A previous study has shown that an important factor for education satisfaction is whether the education is useful or relevant to their job [2]. Because the education results in a vocational degree, it is essential for the students to be prepared, and for the university to prepare the students, for their professional working life. In addition, it is important for the students to be able to develop their professional identity to facilitate the transition from pharmacy student to pharmacist [7,18]. The master’s graduates generally felt more prepared to work as pharmacists compared with the bachelor’s graduates. This is not surprising because the master’s graduates already have a bachelor’s degree and, in many cases, have been working, for example in community pharmacy, prior to being admitted to the master’s program. With work life experience, novice practitioners develop professional identity and learn from more experienced practitioners by social interactions [19]. One challenge reported with distance education is developing a professional identity [20]. However, in the present study a majority of the graduates agreed or strongly agreed that the program had given them a clear professional identity. Educational activities that support the development of a professional identity are important in a vocational degree, including curricula and education design [21,22], reflective portfolios [23,24,25,26], and interprofessional learning [27]. Internships (introductory and advanced pharmacy practice experience (IPPE and APPE, respectively)) and integration of theory and practice are important factors to students’ formation of professional identity [21]. At Umeå University, reflective professional development portfolios have been implemented in order to address the formation of a professional identity [23]. Mentor discussions have also been introduced where the students have the opportunity to meet with pharmacists working within different fields, for example community pharmacy, hospital, and pharmaceutical industry. Activities involving interprofessional learning are in place within the medical faculty but have not yet involved the pharmacy programs.

The analysis showed that graduates who stated that the program gave them a clear professional identity were more satisfied with their job and thought that the duties at work reflected their education. The results suggest that, if the education can provide a clear professional identity, the graduates will experience higher job satisfaction. Having a strong professional identity will enable the graduates to better identify themselves as pharmacists which, in turn, will make them experience a higher satisfaction with their work and their professional role. However, only 64 of 94 graduates answered the question regarding professional identity. This may be due to uncertainty among the graduates as to what a ‘clear professional identity’ actually means. Professional identity was not defined in the survey, it is therefore the respondents’ own interpretation they have related to when answering the question. If the respondents who did not answer the question had answered no, the results may have been different. Furthermore, the size of the two groups, i.e., those who believed that the education had given them a clear professional identity compared to those who did not believe this, was very different which may have affected the results. A previous study in Canada showed that community pharmacists lack a clear professional identity, and this may affect pharmacists´ interactions with other healthcare personnel or patients [7]. Another study concluded that pharmacy students experienced challenges when forming their professional identity and supporting the formation of a professional identity is an important goal for pharmacy education [18].

The graduates in this study participated in the survey one to four years after graduation. Asking about education satisfaction in retrospect may be an advantage; the graduates have a better ability to reflect on their education and it may be easier for the graduates to assess their education in relation to their current job situation [11]. The graduates were asked whether they, a year or a couple of years after graduation, had changed their perception of the value of their education compared to immediately after graduation. In total, one-third answered that they were very much more or slightly more positive. Reasons for this were not explored in detail. However, the graduates were given the opportunity to comment on their answer and these anecdotal comments were used to describe the respondents’ views. In these comments, the graduates mentioned that after graduation, they had realized the importance of their work and their role as pharmacists. Among the graduates who answered that they were very much or slightly more negative, the comments generally related to the commercialization in the pharmacy market and that they did not feel that their competence was appreciated. The risk of an increased commercialization, where the primary focus is on sales, is reduced time for counselling which is an essential part of the pharmacy profession [12]. It appears that, generally, the change in the perception of their education, whether in a positive or negative way, is related to their work experience as pharmacists. Perhaps for some graduates, the expectations did not match the reality in the workplace. Such disparities have previously been reported among pharmacists [28,29].

Web-based education has been shown to provide flexibility to combine studies with family or work commitments and the possibility to commence higher education without having to move [30,31,32,33,34,35]. Flexibility is often considered to be a major reason for choosing a web-based program [34,35]. Distance education generally attracts older, female students [30,36], which is also reflected in the gender distribution of students in the pharmacy programs at Umeå University [34,37]. The main reason for choosing to study pharmacy at Umeå university was that the education was offered online, suggesting that the graduates were actively looking for a web-based option. Campus-based pharmacy education is offered at other universities in Sweden.

Regarding the various educational activities, the graduates participating in the survey believed that, in particular, pharmacy practice, lectures, seminars, and problem-based learning were of great or very great usefulness after graduation. However, activities such as laboratory experiments and role play were considered less useful. This might reflect their current job assignments. A majority of the graduates participating in the survey work in community pharmacies, thus, it is not surprising that pharmacy practice as an educational activity is considered useful. The graduates in this study did not consider role play especially useful, which may be due to the circumstances for which role plays are conducted. Role play is commonly used in communication training and is an example of active learning. Studies have reported that role play has a positive effect on students’ learning and development of communication skills [38,39,40,41,42]. The success of role play often depends on creating a realistic and authentic setting. One way to further develop role play activities in the pharmacy programs could be to use actors to play the counterpart instead of fellow students or university teachers. Another alternative is the use of virtual worlds. Previous studies have shown that virtual worlds have the potential to create realistic learning experiences [43,44]. These have been perceived by pharmacy students as an attractive way to train communication since they provide an authentic and safe environment [45,46]. The use of many different approaches is often beneficial in developing communication skills, and scaffolding increases this effect [47,48,49].

During their education, students are given several possibilities to provide feedback on their academic studies and educational activities using course and program evaluations, for example. Based on these evaluations, updates to the course material and implementation of new educational activities and assessment methods occur regularly. Besides this, alumni surveys are also conducted on a regular basis, in which graduates can look back on their education and provide valuable insights. The feedback, provided in these different ways, all contribute to improve curricula and course design.

A majority of the graduates were female, reflecting the gender division in the pharmacy workforce in Sweden, as well as for pharmacy students in Sweden and other countries [36,50,51,52,53]. Age and gender of the respondents were similar for all graduates, as can be shown by comparing with data from the university’s administration system; this indicates that this was a representative sample with respect to age and gender.

This study has some strengths and limitations. One strength is that it has been possible to study education satisfaction over time by comparing the results from two groups of graduates. Furthermore, asking graduates about education satisfaction some time after graduation is an advantage as they can then also relate their education to their professional life. However, the generalizability of the results is limited as the study only explores education satisfaction at one university and within one academic subject. It would for example be interesting to explore education satisfaction after web-based and campus-based education. Furthermore, graduates who are more positive towards their education may be more likely to complete the survey compared to those who are more negative; selection bias can therefore not be excluded. Reminders were not sent which may have limited the response rate. A definition of professional identity was not included in the survey and therefore the responses were based on the respondents’ own interpretation of the term. To improve the survey for future use, a definition of professional identity should be included. Factors important for education satisfaction would be interesting to study further in order to provide educators with better tools to develop the programs.

## 5. Conclusions

Education satisfaction among pharmacy graduates in this study was high. A majority of the respondents felt that their studies gave them a clear professional identity and that they felt prepared to work as pharmacists after completing the program. Comparison of the results with a previous survey showed that education satisfaction has been maintained over a relatively long time (2006 to 2018). An association was found between professional identity and job satisfaction suggesting that if the programs can provide a clear professional identity, the graduates will experience higher job satisfaction. Exploring education satisfaction among graduates may help educators to further develop the programs, and aid in work with recruitment strategies. Furthermore, it will enable educators to better prepare the students for their professional working life by developing their professional identity during their studies.

## Figures and Tables

**Table 1:** Questions included in the data analysis.

Questions *
1. I felt well prepared to work as a pharmacist after completing the program
2. I am very satisfied with the program
3. The program gave me a clear professional identity
4. I believe that the students’ opinions were considered important by the teachers
5. The knowledge and skills acquired during the program are useful in my current job
6. I would recommend the program to a prospective student
7. My duties at work reflect the program
8. If I were to choose my career today, I would choose pharmacy again
9. If I were to choose today, I would start the same education
10. All things considered, how often are you satisfied with your job

* Questions 1–6 were reported on a five-point Likert scale (strongly disagree (1); disagree (2); neither agree or disagree (3); agree (4); and strongly agree (5)). These questions were dichotomized to disagree (Likert responses point 1–3) and agree (Likert responses point 4–5). Question 7 had yes and no as options. Question 8 was dichotomized to no (definitely no and don’t know) and yes (definitely yes and maybe). Question number 9 was dichotomized to yes (yes, the same education at the same university) or no (yes, the same education but at a different university; no, another education at the same university; no, another education at another university and no, I would not start any education). Question 10 was dichotomized to not satisfied (those who responded never or rarely and sometimes satisfied) and satisfied (those who responded satisfied most of the time and satisfied all of the time).

**Table 2:** Respondents participating in the survey in 2019 (graduation year 2015–2018). For comparison, age and gender of all graduates from the programs have been retrieved from the university’s administration system.

Characteristics	Graduates Bachelor’s Degree *N* (%) *N* = 63	Graduates Master’s Degree *N* (%) *N* = 31 *	Graduates All Degrees *N* (%) *N* = 94
Gender (alumni survey) Women			
	61 (96.8)	26 (83.9)	87 (92.6)
Gender (university administration system) Women	(*N* = 150)	(*N* = 72)	(*N* = 222)
	138 (92.0)	61 (84.7)	199 (89.6)
Age (alumni survey) MeanStandard deviation Range		(*N* = 30)	(*N* = 93)
	34.3	36.3	35.0
	9.2	7.0	8.6
	24–62	26–55	24–62
Age (university administration system) Mean Standard deviation Range	(*N* = 150)	(N = 72)	(*N* = 222)
	33.4	37.0	34.5
	8.3	7.8	8.3
	24–62	26–60	24–62
Country of birth Sweden Outside Sweden	(*N* = 62)	(*N* = 30)	(*N* = 92)
	51 (82.3)	20 (66.7)	71 (77.2)
	11 (17.7)	10 (33.3)	21 (22.8)
Current employment		(*N* = 30)	(*N* = 93)
Community pharmacy	51 (81.0)	20 (66.7)	71 (76.3)
Hospital pharmacy	4 (6.3)	0 (0.0)	4 (4.3)
County council **	2 (3.2)	4 (13.3)	6 (6.5)
Pharmaceutical company	0 (0.0)	4 (13.3)	4 (4.3)
Dose dispensing pharmacy	3 (4.8)	0 (0.0)	3 (3.2)
University	1 (1.6)	2 (6.7)	3 (3.2)
Municipality	2 (3.2)	0 (0.0)	2 (2.2)

* Overall, 13 of these graduates also had a bachelor’s degree from Umeå university. These graduates are counted as graduates with a master’s degree in the analysis, i.e., the highest degree is considered. ** County councils are responsible for the public health care systems in Sweden.

**Table 3:** Questions regarding education satisfaction among pharmacy graduates (graduation year 2015–2018). Percentage of graduates choosing the different options in the survey.

	Number and Percentage of Graduates *N (%)*				
Question	Strongly Disagree	Disagree	Neither Agree Nor Disagree	Agree	Strongly Agree
I felt well prepared to work as a pharmacist after completing the program (*N*= 92)	2 (2.2)	6 (6.5)	9 (9.8)	49 (53.3)	26 (28.3)
I am very satisfied with the program (*N*= 93)	0 (0.0)	1 (1.1)	3 (3.2)	54 (58.1)	35 (37.6)
The program gave me a clear professional identity (*N* = 64)	1 (1.6)	0 (0.0)	4 (6.3)	30 (46.9)	29 (45.3)
I believe that the students’ opinions were considered important by the teachers (*n* = 62)	0 (0.0)	4 (6.5)	13 (21.0)	33 (53.2)	12 (19.4)

**Table 4:** Multivariate logistic regression including different questions regarding education satisfaction. Graduation years 2006 to 2014 and 2015 to 2018. Results presented show the number and percentage of students answering agree or strongly agree * or yes ** to the questions.

Question	Graduation Year 2006–2014 *N* (%)	Graduation Year 2015–2018 *N* (%)	Odds Ratio ^1^	95% Confidence Interval	*p*-Value
The knowledge and skills acquired during the program are useful in my current job *	192/218 (88.1)	76/92 (82.6)	0.672	0.334–1.351	0.265
I would recommend the program to a prospective student **	197/214 (92.1)	86/93 (92.5)	1.057	0.412–2.712	0.909
My duties at work reflect the program *	162/220 (73.6)	67/93 (72.0)	0.994	0.567–1.743	0.984
If I were to choose my career today, I would choose pharmacy again **	185/218 (84.9)	75/93 (80.6)	0.778	0.404–1.498	0.453

^1^ Controlled for age.

**Table 5:** The graduates’ attitudes towards the usefulness of various educational activities included in the program (graduation years 2015 to 2018).

Educational Activity	No or Little Usefulness *N* (%)	Great or Very Great Usefulness *N* (%)	Don’t Know *N* (%)
Group work	30 (31.9)	61 (64.9)	3 (3.2)
Individual assignments	19 (20.4)	71 (76.3)	3 (3.2)
Internet based systems	15 (16.1)	50 (53.8)	28 (30.1)
Laboratory experiments	53 (56.4)	39 (41.5)	2 (2.1)
Lectures	11 (11.8)	80 (86.0)	2 (2.2)
Oral presentations	26 (28.0)	65 (69.9)	2 (2.2)
Pharmacy practice	9 (9.7)	82 (88.2)	2 (2.2)
Problem based learning	8 (8.6)	74 (79.6)	11 (11.8)
Role play	40 (43.0)	44 (47.3))	9 (9.7)
Seminars	14 (15.2)	74 (80.4)	4 (4.3)
Written presentations	21 (22.6)	69 (74.2)	3 (3.2)
